# Health-Care Professionals Amid the COVID-19 Pandemic: How Emotional Intelligence May Enhance Work Performance Traversing the Mediating Role of Work Engagement

**DOI:** 10.3390/jcm10184077

**Published:** 2021-09-09

**Authors:** Martin Sanchez-Gomez, Max Sadovyy, Edgar Breso

**Affiliations:** Department of Evolutionary, Educational, Social Psychology and Methodology, Universitat Jaume I, 12071 Castellón de la Plana, Spain; max.sadovyy@uji.es

**Keywords:** SARS-CoV-2, health-care professionals, emotional intelligence, work performance, work engagement

## Abstract

Upon the eruption of COVID-19, frontline health-care workers confronted substantial workload and stress along with braving additional difficulties when performing at work. The main aim of this research was to assess the mediating role of work engagement in the direct impact of emotional intelligence on health-care professionals’ work performance. A cross-sectional study was conducted in several Spanish hospitals during the second half of 2020. A total of 1549 health-care workers (62.1% women; mean age 36.51 years) filled the Wong and Law Emotional Intelligence Scale, the Utrecht Work Engagement Scale and the Individual Work Performance Questionnaire. Our findings demonstrated that work engagement plays a mediating effect between emotional intelligence and work performance, even when accounting for sociodemographic variables. Indeed, among the three constructs of engagement, vigor dimension (a1b1 = 0.09; CI: 0.06; 0.12; *p* < 0.01) emerges over dedication (a2b2 = 0.083; CI = 0.05, 0.1; *p* < 0.01) and absorption (a3b3 = 0.047; CI = 0.02, 0.07; *p* < 0.01) as the most decisive one. Herewith, it is apparent that professionals with a higher self-perception of emotional intelligence report stronger levels of engagement, thereby leading to greater performance overall. The present work evinces the necessity for proactively developing the emotional competencies of the health-care workforce, especially in high-emotional demand contexts.

## 1. Introduction

Mankind is at present poised upon the menace of a newly arisen form of severe acute respiratory syndrome (SARS) specifically referred to as SARS-CoV-2 [1]. The designation of the currently known coronavirus disease (COVID-19) surfaced in December 2019 after the first single patient case in Wuhan, China, thereby gradually depicting a global health-care urgency that manifests itself daily across all types of repercussions on population while aggravating the interlinked susceptibility of global connectedness [2]. By virtue of such a nexus, our internal capacity to balance ourselves is endangered and thus jeopardized by these outer triggers and, subsequently, the short or long lasting experience of being exposed to them might: (a) lead to an inward state of stress, which, while functioning as an adapter under strenuous circumstances, may: (b) escalate into the arousal of emotions such as fear and anxiety when endured for a sustained length of time [3]. Thenceforth, an attempt is undertaken in the present paper to provide visibility to front-line health-care workers in their struggle to safeguard our lives by endangering theirs, as well as to reveal the critical issues for enhancing public mental health resources through the identification of core components that can promote survival rates, physical condition and mental welfare as human beings submerged in such an era of global unstableness [4].

### 1.1. Health-Care Professionals in a Front Line Context

Health-care professionals confront a profound apprehension not only when exposed to stressors such as the consolidation and sustenance of the health condition of their patients, but also in terms of struggling with enormous stress and fear coupled with a sense of social stigma bound to their occupational position [5]. Above the aforementioned issues, the COVID-19 professional context arises as a scenario characterized by several negative consequences over health-care workers, especially in concern to nuisances associated with mental health [5]. It is indeed for this purpose that health-care must be comprised both in terms of the prevention of pathology, in addition to corporal, cognitive and public welfare, both imperatives representing an authentic challenge in such an increasingly volatile era health-care workers are constantly striving for combating in order to guarantee: (1) the survivability of its largest achievable population and (2) an enhanced resilience against upcoming resembling scenarios [6]. Globally extrapolated and frequently at grave individual hazard, health-care professionals in almost every country have been devotedly toiling on the front lines treating COVID-19 victims, but have not at all times garnered the recognition they are entitled to regardless of their substantial interventions [7]. Over and above, primary health-care personnel run a correspondingly heightened risk of disease, along with incurring unfavorable emotional responses through apprehension, avoidance of contagion or a sense of helplessness positioning them at an additional frontline characterized by emotional vulnerability towards such a relentless worldwide health-care emergency [8,9].

### 1.2. Emotional Intelligence as an Inner Resource

The negative relationship between such an emotionally demanding COVID environment and health-care professionals’ outcomes could be explained by the health impairment process of the job demands-resources theory (JD-R) [10]. According to this approach, specific work characteristics (i.e., job demands and job resources) are associated with work outcomes (e.g., engagement, well-being, work performance). Thus, high job stressors such as the COVID-19 pandemic may generate a sense of exhaustion in employees’ mental and physical resources, subsequently lowering their energy levels and leading them to health issues. Suitably, one recognized and validated resource that safeguards the psychological quality of humans is identified as the ability to be emotionally intelligent. Accordingly, emotional intelligence (EI) is portrayed as the capability to perceive, facilitate, understand and manage one’s own and others’ emotions [11]. Therefore, it might be worth deeming that health-care professions have been described as an emotional practice, accurately categorized as a form of emotional labor [12]. Hence, alongside with the specialized expertise requirements, modern clinical praxis encompasses a holistic continuum of action as well that embraces the emotions, relationships and values of patients whose holistic care at the hands of health-care workers owes a commitment as empathizers [13]. Therefore, taking into consideration the JD-R theory, EI stands to be an imperative proficiency for the health-care workforce in managing both self-emotions whilst rendering the others, namely in the presence of the evidence from former findings of research indicating the beneficial influence of an emotionally intelligent health-care professional with enhanced levels of service provision quality, self-reported sense of welfare and empowerment at the workplace [14]. Thence, EI traversing the ability approach is esteemed both as a self-psychological appeal and a forecaster of performance, yet absence thereof might attract a harsh forthcoming aftermath particularly in such present scenario humanity is currently absorbed [15,16].

### 1.3. Health-Care Professionals’ Work Performance Amid COVID-19

Under this scope of abduction, professional sectors are presently imperiled by a pervasive phenomenon that not only symbolizes a mental health crisis [17], but bewilderment for any corporation as to ensure the occupational integrity as well as the versatile labor efficiency of its internal departments [18]. Inwardly, the core of work performance consists of three major domains: (1) task performance, i.e., on-task activities straightforwardly pertaining to the particular profile role; (2) contextual performance, i.e., actions that transcend the duties and functions of the assigned occupation; and (3) counterproductive work behavior, i.e., intended detrimental attitudes that hinder the organizational norms and practices of its constituents [19]. Under this suite of constructs, work performance is referred to herein as the total accomplishment of the employees in compliance with the organizational requirements in terms of time, method and chores [20]. Accordingly, certain premises concerning the inherent complex dynamics of health-care services imply that: (a) unpredictable strains and requests demand pliability and prioritization; (b) unreliable policies in envisioning competing interactions involve demands that disrupt workflow; and (c) secure coping with emotional constraints is indispensable to shield health-care procedures [21]. In due time, not merely must the reaction of public health to COVID-19 alleviates incertitude by proffering accurate statistics, but equally convert this challenge into an investment in mental health expertise, thereby enhancing the abilities of health-care professionals to competently navigate emotions in turn insuring quality of care in the course of their formally designated duties [22].

### 1.4. Engagement as a Mediator

At the heart of it, raising self-awareness of personal emotions along with actively providing their insights and first-hand stories with their patients may assist them in retaining centeredness amid this pandemic, both for the maintenance of the desired quality of their performance and to forth-propel it via the intensity of their subsequent engagement at work [23]. In other words, engagement is a multistranded construct concerned with occupational environment, individual and labor assets as well as its exigencies peaking with variables of demography, therefore being characterized as a pleasant and fulfilling mental condition associated with the professional occupation expressed under three facets: (1) vigor, i.e., the eagerness to pour energy; (2) dedication, i.e., the enthusiasm to be involved and socially proactive; and (3) absorption, i.e., the tendency to lean into a sharpened approach and attentiveness [24]. By the same token, work engagement acts as a compensatory agent between the impression of achievement on completion of a certain duty and the apprehension of disappointment at the time of its execution, underscoring the fact that this perceived exasperation when low permits an increment in performance by means of such a needed and aforementioned work engagement degree [25]. Categorically, insofar employees are cognizant about managing own organizational work reactions by responding appropriately and behaving in manners that nurture improved peer and supervisor interactions, this fact may prompt them to exert higher arousal and become more energetic and prideful at work [26,27]. Hence, determining that EI has the potential to alter self-perceptions and attitudes toward a diverse spectrum of occupational phenomena (i.e., heightened EI may elicit the active use of assertive co-decisional techniques to contend with hostile peers versus positively portraying workplace pressures) and thus interpreting their brighter conditions at work as more rewarding because of their self-proactive approach to their professional responsibilities [28]. Essentially, work engagement over time proved a mediated ambience of clinical practice, declined adverse events and a such a heightened rate of patient safety to the extent of alleging that a well-engaged hospital workforce is crucial at identifying determinants of preventable occupational disorders as to contrive countermeasures, paradoxically in an era of constant tension that barely has the luxury of competing with all these diversified and seemingly never-ending demands [29]. Therefore, so as for that, the aforementioned JD-R theory posits that EI shall be considered as a personal resource that may enhance the health-care professionals’ engagement, which in turn could buffer their performance levels by reducing some of the COVID-19 negative aftereffects.

### 1.5. The Present Study

**Figure 1:**
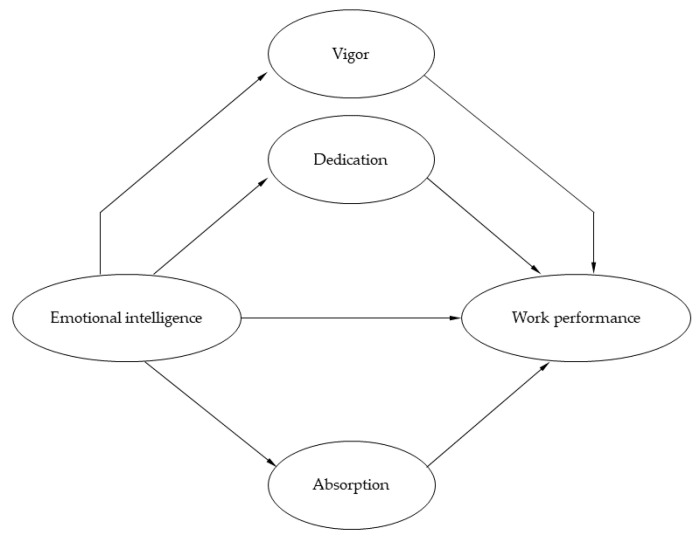
Proposed model of work engagement dimensions mediating the relationship between emotional intelligence and work performance.

Following the preceding theoretical and empirical findings, the aim of the present investigation was to provide additional empirical support for the potential underlying mechanism in the relationship between EI and work performance of health-care professionals amid the COVID-19 pandemic. In particular, we propose three hypotheses: EI would be positively correlated with health-care professionals’ engagement (H1a) and work performance (H1b); work engagement (vigor, dedication and absorption) would be positively correlated with health-care professionals’ work performance (H2); and work engagement (all vigor, dedication and absorption dimensions) would mediate the relationship between EI and work performance (H3). The hypothesized model is drawn in Figure 1.

## 2. Materials and Methods

### 2.1. Sample

By means of utilization of a cross-sectional design, a descriptive study was conducted in several Spanish hospitals during the second half of 2020 in order to examine the mediating role of the three engagement dimensions in the relationship between EI and work performance in a sample of health-care professionals. The sample included 1549 people (62.1% were women). The mean age was 36.51 years (SD = 12.47, range = 20–67 years), the overall work experience was 8.71 years, and the average organizational seniority was 6.1 years. As concerns the educational level of the participants, 2.6% had high school, 19.4% vocational education, 32.4% equal or more than 4 years of college and 45.6% more than 4 years of college. In relation to the marital status of the participants, 34.2% were married, 50.8% single or in a relationship, 13.1% separated/divorced and 1.9% widowed. Participants exclusively accounted for the health-care professional category constituted by doctors (26.9%), nurses (43.4%), porters (17.2%) and several minor professions in hospitals (e.g., psychologists and physiotherapists among others) which represented the remaining 12.5%.

### 2.2. Instruments

#### 2.2.1. Emotional Intelligence

The Wong and Law Emotional Intelligence Scale (WLEIS) in its Spanish adaptation was utilized to address the perception of EI [30]. This scale has exhibited reliable psychometric properties [31]. The WLEIS is a self-report itemized evaluation containing 16 items with a 5-point Likert scale. Extensive factor configuration investigations have identified four composite domains formed by four items apiece: evaluation of one’s own emotions (SEA; “I have a good sense of why I have certain feelings most of the time”), evaluation of the emotions of others (OEA; “I always know my friends’ emotions from their behavior”), use of emotions or assimilation (UOE; “I always set goals for myself and then try my best to achieve them”), and regulation of emotions (ROE; “I can always calm down quickly when I am very angry”). The internal consistency of each of these constructs was satisfactory: SEA (0.85), OEA (0.81), UOE (0.86) and ROE (0.90).

#### 2.2.2. Work Performance 

The Individual Work Performance Questionnaire (IWPQ) in its Spanish adaptation [32] was employed to evaluate work performance [33]. The IWPQ integrates an 18-item scale aimed at scoring the three core dimensions of work performance: task performance (“I kept in mind the results that I had to achieve in my work”), contextual performance (“I started new tasks myself, when my old ones were finished”) and counterproductive work behavior (CWB; “I focused on the negative aspects of a work situation, instead of on the positive aspects”). Each item was indicated along with a five-point Likert scale (0 = seldom to 4 = always for task and contextual performance; and 0 = never to 4 = often for CWB). On the basis of earlier findings [34] as well as with the intention of capturing the completeness of the notion of work performance, it was ultimately deemed necessary to parse both the positive and negative metrics to provide an all-in-one score that encompassed all three dimensions. For this conversion to function, it was thereby imperative to invert the negative sense of the CWB rating and thus enabling the consolidation to function as one. The internal consistency is detailed in Table 1.

#### 2.2.3. Work Engagement

The Utrecht Work Engagement Scale [35] was administered to measure work engagement. A well-approved Spanish version of the scale was selected [36]. This tool is composed of nine items based on a 7-point Likert-type scale ranging from 0 (never) to 6 (always). This scale gauges the three dimensions of work engagement: vigor (e.g., “At my work I always persevere, even when things do not go well”), dedication (e.g., “I find the work that I do full of meaning and purpose”) and absorption (e.g., “I feel happy when I am working intensely”). The internal consistency is detailed in Table 1.

### 2.3. Procedure

A self-administered socio-demographic questionnaire was devised to collect data of age, gender, marital status, educational level, professional sector, work experience and organizational seniority. In accordance with a convenience sampling approach and in line with former research, the sample was compiled with the collaboration of undergraduate students who were familiar with the administration of questionnaires. Upon the investigators’ guidelines, the students subsequently contacted several hospitals, querying the human resources area about the possibility of cooperating. Additionally, an e-mail invitation to the experiment was sent to 4322 people, 1588 of whom responded (response rate = 36.9%). Out of all the responses collected, 39 participants were discarded due to omission of the solicited data. This entire procedural design was performed under the terms of the criteria provided by Wheeler et al. [37] for the implementation of this typology of sampling method. The questionnaire was completed online using the Google Forms platform during the first half of 2021. It is noteworthy to mention that this research adhered to the ethical guidelines referred to in the Declaration of Helsinki and was endorsed by the ethics committee of the corresponding entity to which this article pertains. Prior to joining the study, participants registered that they were over 18 years of age and were instructed about their voluntary involvement and the confidentiality of the data. To ascertain their privacy, a statement was provided in the administered questionnaires concerning anonymity and that their results would be treated exclusively for research aims. Data collection was securely documented in a database under the control of the principal researcher in charge of the conducted statistical analysis.

### 2.4. Data Analysis

The analyzed data were processed using the SPSS software version 26.0 (IBM). The first analysis entailed descriptive statistics, encompassing the mean, standard deviation and reliability of the study variables. To test the hypotheses H1a, H1b and H2, Pearson correlations were conducted between EI, work engagement and work performance. These analyses were necessary before testing the mediator model. After that, H3 was tested by means of a structural equation modeling (SEM) approach using AMOS 26 (IBM). Goodness of fit was controlled through the Chi-square index (*χ*^2^), the comparative fit index (CFI), the Tucker-Lewis index (TLI), the root mean square error of approximation (RMSEA), and the standardized root mean square residual (SRMR), as proposed by Baron and Kenny [38]. Furthermore, in order to determine the moderating effect in the proposed model, multiple mediation analyses were conducted. This technique uncovered the effects of each EI dimension (predictor) by means of five distinct routes for each branch of EI and total EI. An indirect pathway is statistically significant if the associated 95% confidence interval (CI) does not include zero. For this objective, the PROCESS 3.3 macro [39] was deployed. Tracing a bootstrap method with 10,000 data samples, which renders 95% bias-corrected confidence intervals, it was feasible to scrutinize conditional models for predicting direct and indirect effects between variables. To ascertain the relative magnitude of specific indirect effects, contrasts were estimated using bias-corrected and accelerated bootstrap intervals. The sociodemographic variables were controlled to avoid possible interference effects.

## 3. Results

### 3.1. Descriptive Analyses of Emotional Intelligence, Work Engagement and Work Performance of Front-Line Health-Care Workers

The first analyses were designed to describe correlations, means, standard deviations, and reliabilities concerning the study variables (Table 1).

Regarding H1a, as shown in Table 1, EI correlated significantly with all engagement variables (vigor: *r* = 0.39; dedication: *r* = 0.33; absorption: *r* = 0.32). On the other hand, EI correlated with all work performance variables (task performance: *r* = 0.42; contextual performance: *r* = 0.36; counterproductive work behavior: *r* = −0.25) as well as with the overall score named work performance (*r* = 0.48), thereby supporting H1b. As hypothesized (H2), all three engagement dimensions were significantly correlated with work performance, being this relationship positive-correlated with task and contextual performance, but negatively correlated with CWB. The Cronbach’s alpha values showed good reliability of the study variables (between 0.77 and 0.90).

### 3.2. Multiple Mediation Analyses

**Figure 2:**
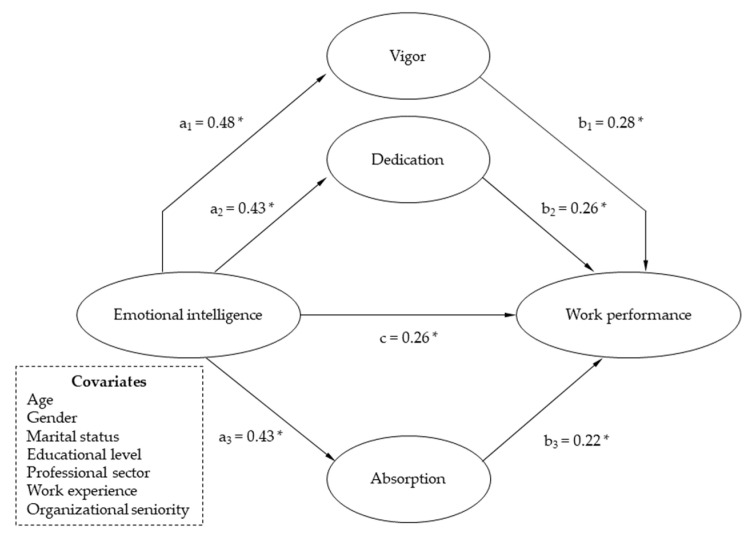
Multiple mediation model of the engagement dimensions acting between EI and WP. * *p* < 0.01.

Regarding H3, SEM analyses allowed to examine the association between EI and work performance through engagement. The results show an excellent fit (*χ*^2^ = 3.862, CFI = 0.995, TLI = 0.990, RMSEA = 0.012, SRMR = 0.019). In order to analyze the specific direct and indirect effects in the relationship between health-care professionals EI and work performance, an analysis of mediation was conducted. Moreover, this analysis offered the possibility of delineating the respective functions between the three domains of engagement. The confidence intervals (CIs) were computed utilizing a multiple mediation modeling approach. Table 2 reports the findings of the indirect impacts along with their 95% CIs. It is essential to assert that each covariate (i.e., age, gender, marital status, educational level, professional sector, work experience and organizational seniority) exhibited a significant influence. As depicted in Figure 2, the bootstrap calculation disclosed the significant direct effect of EI toward work performance (*c* = 0.26; *p* < 0.01). Upon computation of the indirect effects (Table 2), vigor (Path 1), dedication (Path 2) and absorption (Path 3) demonstrated a significant indirect effect (a1b1: vigor indirect effect = 0.091; 95% CI = 0.06, 0.12; a2b2: dedication indirect effect = 0.083; 95% CI = 0.05, 0.11; a3b3: absorption indirect effect = 0.047; 95% CI = 0.02, 0.07), as it can be noticed at Figure 2. Probing cross-sectional variances across the three constructs of engagement, vigor yielded the largest impact, suggesting that this component is of major concern as a mediator within the relationship between EI and work performance. Determinedly, after accounting for the interaction of the impact of the various covariates, vigor, dedication, and absorption partly mediated the connection between EI and WP. All three of the engagement dimensions and covariates were explanatory of 41% of the variation on work performance (R^2^ adj = 0.41; *p* < 0.001). Post-hoc analyses were subsequently completed so as to identify the similar model for every dimension of EI. The results revealed a resembling structure to that previously documented one in turn enabling to assert that each EI construct and its association with performance are mediated through each and every element of work engagement.

## 4. Discussion

The aim of this investigation sought to examine the mediating enactment of work engagement onto the nexus between EI and work performance of health-care professionals at the height of the COVID-19 pandemic. For this purpose, our results are in line with previous studies [24] by indicating the significant relationship between EI and health professionals’ engagement in all its dimensions (H1a), as well as the positive relationship between EI and performance (H1b). Then, aligned with prior research [27], work engagement reported a positive relationship with health professionals’ work performance (H2). Ultimately, the present paper provides support for a potential mediating effect of engagement on the relationship between EI and job performance (H3).

Subsequently, the present study may provide insight on the mediating role of work engagement as a mediator in the interaction between EI and work outcomes on behalf of such a health-care workforce humankind reposes the utmost faith on a quotidian reliance on [40]. According to the derivative evidence [25], the obtained findings reported in the current article have yielded significant associations for the direct interaction between EI and work performance crosswise to the mediating role of work engagement. Crucially, the mediation analyses exhibited that work engagement as a mediator and its cognates are significantly connected to EI and work performance. Similar to former investigations, a high level of engagement is related to satisfactory performance construed via the perception of success in fulfilling occupational assignments [27]. Hence work engagement mediates health-care professionals’ self-appraisal of their mental workload, as well as oral and visual aptitude, while mismanaged emotional reactions such as frustration appear to diminish their performance across a narrower extent of work engagement [25]. At the bottom line, a solid approach for maintaining focus during this pandemic in terms of performance quality and to sustain it through the depth of engagement thereafter was based on cultivating personal awareness of feelings and proactively relaying perspectives of personal experiences encountered first-hand with patients [23].

Therefore,, there are professionals with heightened EI who may leverage their affective capabilities to recognize pandemic-related reactions (e.g., anxiety, worry or uncertainty), which may encourage the agency to adopt emotional responses to ameliorate their discomfort in turn achieving their occupational effectiveness [15]. These assertions concur with prior comparable empirical evidence on the mediating influence of work engagement inasmuch as EI may serve as an enabler in accounting for workers’ functioning when it comes to comparing individuals with less EI and those with more of it being both groups mediated by their degrees of work engagement conjoined with their work performance thereupon [26,28]. Accordingly, greater EI indices may assist professionals in maximizing their potential in regard to how they feel at work and alongside the continuum aid in steadying and preserving the depth and breadth of their work performance in such a scenario of fluctuating emotional conditions [14]. 

On balance, work engagement may be benchmarked as a mediator insofar as the current research has been in conformity with that approach in its bid to integrate the pandemic into a prospective insight towards the relevance of engagement at work [23,24]. Upon this categorization, it can be contended that the interactions encountered throughout the course of the mediating analyses have revealed adequate credibility. Hence, it may in turn be forecasted that the submitted results might lead a novel course in which further research shall be pursued in order to buttress the insights and per se the knowledge that has emerged from the herein presented literature. Fundamentally, those individuals who responded with higher levels of EI seem to hold more resources to display superior work performance owing to the mediating role of work engagement as opposed to those whose emotional capability stood at a baseline position and, consequently, delivered diminished work performance on the basis of decreased rates of work engagement. On a conclusive note, it appears that greater levels of EI permit health-care professionals to benefit from a stronger occupational performance by committing further at work with the ultimate purpose of feeling self-fulfilled and maintaining economic stability in times of daily exigencies and uncertainties. Ultimately, these goals tend to be compatible with the aforementioned JD-R theory by positioning the EI role as a fundamental personal resource in stressful contexts such as the current one [10].

Conclusively, this investigation helps empower experiential proof on the major mediating effect of work engagement in times when the COVID-19 pandemic entails a genuine hazard to all occupational streams within the health-care workforce and the subsequent job performance at it. Indeed, harnessing their labor and talents converts them into the most critical endowment for organizations to conserve and upgrade their status in the field [41]. This approach might facilitate an improved management of emotions and, ultimately, lead to increased work engagement rates sustaining in turn the stability of their occupational efficiency [42].

### Limitations and Future Research

Certain caveats are indicative of potential thematic areas of concern for further exploration in this paper. At the outset, it is critical to remark that the employment of cross-sectional data hampered the operation of deciphering the extent of the associations between the variables as well as their orientation. Yet, the findings of the herein presented research are substantiated by a solid and robust scientific evidence baseline. Still, replicating these outcomes with longitudinal methods in this study might provide further insight into the mediating role of work engagement in the correlation between EI and work performance.

An additional restricting factor stems from the evaluation approach adopted for the appraisal of EI. Namely, from the determination to be advantageous in terms of providing a speedier administration and thus the choice of the WLEIS, it is hereby borne in mind that this as a self-report questionnaire and it is recommended to employ both self-reports and performance tests when attempting to use it for the measurement of EI [43,44]. Furthermore, in conformity with prior investigations that screened both incremental and predictive feasibility [45], aptitude EI scores such as MSCEIT [46] or MEIT [47] are deemed more suitable to introduce regardless of the WLEIS-fact to be widely implemented, since its assessment is grounded on the domain of the original model of EI [11].

Ultimately, the unsettled emotional climate in those periods of severe conditions in the course of the current pandemic may have altered individuals’ perceived self-image as related to EI, work engagement, and job performance. Indeed, the present scenario depicts a time fraught with uncertain results if hypothesized to publish investigations such as the present one before the occurrence of the actual disease prior to driving human emotions to the borderline [48,49]. 

## 5. Conclusions

Precedent experience in catastrophes, pandemics and other high-trauma events might imply providing further assistance tools for health-care professionals so that they develop and acknowledge their own feelings. Furthermore, sharing their perceptions and lived history with patients can aid them in preserving their competence and awareness within the midst of these severe disruptive conditions. That is due to the reality that health-care is not a mere scientific endeavor, but a subject of empathy and thus communicative techniques are required to convey it. Accordingly, outreach efforts in this arena should prioritize enhancing self-confidence to competently perform clinical assignments, encompassing COVID-19 specific issues, advocating self-protection policies and encouraging health-care workers to be supported by the commitment of empowered leadership. Additionally, building a cooperative internal organizational culture is instrumental in order to ensure adequate levels of emotional intelligence and ultimately higher performance within a superior work engagement in such a day-after-day emotionally self-consuming occupation. As a final concluding statement, the results demonstrated in this investigation evidence the significant direct effect of emotional intelligence toward individual work performance (c = 0.26; *p* < 0.01) as well as the mediating involvement of engagement in a sample of Spanish health-care professionals. Specifically, after controlling for sociodemographic variables, vigor (a1b1 = 0.09; CI: 0.06; 0.12; *p* < 0.01) emerged over dedication (a2b2 = 0.083; CI = 0.05, 0.1; *p* < 0.01) and absorption (a3b3 = 0.047; CI = 0.02, 0.07; *p* < 0.01) as the most decisive engagement dimension. Apparently, professionals exhibiting elevated levels of emotional intelligence dispose of optimal internal resourcefulness when confronting work demanding issues and, as a corollary, do maximize their engagement, which in turn promotes the perception of sustained effectiveness at work and facilitates an improved work performance indicator. Such findings attest to the critical significance of work engagement in comprising work performance in health-care environments and emphasize the role of emotional intelligence as an empowering variable particularly valuable in demanding contexts such as the COVID-19 pandemic. Therefore, the current research reasserts the imperative to develop such constructs in health-care facilities in pursuit of enhancing emotional intelligence to build healthy workplaces that may assist workers in achieving their peak performance when immersed in those enthusiasm-inducing occupational stations.

## Figures and Tables

**Table 1:** Descriptive Statistics and Correlations between Study Variables.

Variables	1	2	3	4	5	6	7	8
1. Emotional intelligence	-							
2. Vigor	0.39 *	-						
3. Dedication	0.33 *	0.78 *	-					
4. Absorption	0.32 *	0.71 *	0.76 *	-				
5. Task performance	0.42 *	0.35 *	0.28 *	0.28 *	-			
6. Contextual performance	0.36 *	0.44 *	0.43 *	0.43 *	0.37 *	-		
7. CWB	−0.25 *	−0.36 *	−0.32 *	−0.26 *	−0.22 *	−0.10 *	-	
8. Work performance	0.48 *	0.56 *	0.52 *	0.49 *	0.67 *	0.76 *	−0.69 *	-
Mean	5.46	4.44	4.68	4.39	3.14	3.07	1.59	2.91
Standard Deviation	0.85	1.09	1.20	1.19	0.61	0.68	0.91	0.50
α	0.90	0.77	0.85	0.79	0.77	0.83	0.79	0.86

Note: *n* = 1549. * *p* < 0.01. CWB = counterproductive work behavior. α = Cronbach’s alpha.

**Table 2:** Multiple Mediating Analyses Testing the Mediating Effect of Engagement Dimensions.

Model Pathways	Point Estimate	SE	Normal Theory Tests			95% Bias-Corrected CI	
			Effect	Z	*p*	Lower	Upper
Total effect	0.221	0.01				0.24	0.29
EI→V→WP	0.091	0.01	0.09	5.89	<0.01	0.06	0.12
EI→D→WP	0.083	0.01	0.08	5.40	<0.01	0.05	0.11
EI→A→WP	0.047	0.01	0.04	3.46	<0.01	0.02	0.07
Model 1: *p* < 0.01; R2 = 0.45; R2 adj = 0.41							
Total effect	0.232	0.01				0.21	0.26
SEA→V→WP	0.104	0.01	0.10	6.70	<0.01	0.07	0.13
SEA→D→WP	0.082	0.01	0.08	5.31	<0.01	0.05	0.11
SEA→A→WP	0.046	0.01	0.04	3.39	<0.01	0.02	0.07
Model 2: *p* < 0.01; R2 = 0.41; R2 adj = 0.37							
Total effect	0.233	0.01				0.20	0.24
OEA→V→WP	0.101	0.01	0.10	6.50	<0.01	0.07	0.13
OEA→D→WP	0.084	0.01	0.08	5.43	<0.01	0.05	0.11
OEA→A→WP	0.048	0.01	0.04	3.52	<0.01	0.02	0.07
Model 3: *p* < 0.01; R2 = 0.42; R2 adj = 0.38							
Total effect	0.234	0.01				0.19	0.24
UOE→V→WP	0.100	0.02	0.10	6.36	<0.01	0.06	0.13
UOE→D→ WP	0.085	0.01	0.08	5.39	<0.01	0.06	0.12
UOE→A→WP	0.049	0.01	0.04	3.50	<0.01	0.02	0.08
Model 4: *p* < 0.01; R2 = 0.40; R2 adj = 0.36							
Total effect	0.228	0.01				0.21	0.25
ROE→V→WP	0.093	0.01	0.09	5.94	<0.01	0.06	0.12
ROE→D→WP	0.081	0.01	0.08	5.25	<0.01	0.05	0.11
ROE→ A→WP	0.054	0.01	0.05	3.94	<0.01	0.03	0.08
Model 5: *p* < 0.01; R2 = 0.42; R2 adj = 0.38							

Note: *n* = 1549. SE = standard error. CI = confidence interval. EI = emotional intelligence. SEA = self-emotion appraisal. OEA = other-emotional appraisal. UOE = use of emotions. ROE = regulation of emotions. V = vigor. D = dedication. A = absorption. WP = work performance.

## Data Availability

The data presented in this study are available on request from the corresponding author. The data are not publicly available due to privacy issues.
